# Structures of the *Shigella flexneri* Type 3 Secretion System Protein MxiC Reveal Conformational Variability Amongst Homologues

**DOI:** 10.1016/j.jmb.2008.01.072

**Published:** 2008-04-04

**Authors:** Janet E. Deane, Pietro Roversi, Carole King, Steven Johnson, Susan M. Lea

**Affiliations:** Sir William Dunn School of Pathology, South Parks Rd, University of Oxford, Oxford OX1 3RE, UK

**Keywords:** T3SS, type 3 secretion system, Mxi, membrane extrusion of invasion plasmid antigens, Yop, *Yersinia* outer protein, type 3 secretion system, *Shigella flexneri*, MxiC, secretion regulation, YopN

## Abstract

Many Gram-negative pathogenic bacteria use a complex macromolecular machine, known as the type 3 secretion system (T3SS), to transfer virulence proteins into host cells. The T3SS is composed of a cytoplasmic bulb, a basal body spanning the inner and outer bacterial membranes, and an extracellular needle. Secretion is regulated by both cytoplasmic and inner membrane proteins that must respond to specific signals in order to ensure that virulence proteins are not secreted before contact with a eukaryotic cell. This negative regulation is mediated, in part, by a family of proteins that are thought to physically block the entrance to the secretion apparatus until an appropriate signal is received following host cell contact. Despite weak sequence homology between proteins of this family, the crystal structures of *Shigella flexneri* MxiC we present here confirm the conservation of domain topology with the homologue from *Yersinia* sp. Interestingly, comparison of the *Shigella* and *Yersinia* structures reveals a significant structural change that results in substantial domain re-arrangement and opening of one face of the molecule. The conservation of a negatively charged patch on this face suggests it may have a role in binding other components of the T3SS.

*Shigella flexneri* is a Gram-negative bacterial pathogen that causes human bacillary dysentery resulting in over a million deaths annually worldwide. The pathogenicity of *Shigella* sp. is dependent on a complex macromolecular machine, the type 3 secretion system (T3SS), that delivers into host cells a set of effector proteins required for invasion. The *Shigella* sp. T3SS consists of structural components of the injection machinery, secreted proteins, chaperones and regulators, all of which are encoded by approximately 25 genes located in the *mxi*, *spa* and *ipa* operons on a large 230 kb plasmid.^1–3^

The delivery of effectors into host cells involves secretion, the crossing of both bacterial membranes via the basal body, and translocation, the passage through the eukaryotic cell membrane. Following assembly of the external needle, the proteins secreted via the T3SS fall into two main categories: translocators and effectors. Upon host cell contact, translocators assemble into the host cell membrane, forming a pore complex, or translocon, that triggers the subsequent export of effectors.^4^ Since translocators must be secreted before effectors, so that effectors will be exported directly into host cells instead of the extracellular milieu, pathogens require mechanisms to ensure hierarchical and temporal control over their secretion. Although the exact mechanisms underlying these processes are not clearly established, several cytoplasmic and inner-membrane proteins have been identified that recognize secretion substrates and respond to specific signals to ensure that structural and sensing components (needle subunits and pore proteins) are secreted first, and that virulence proteins are not secreted before contact with a host cell.

Blockage of effector secretion before host cell contact is mediated, in part, by a protein that has been proposed to act as either a physical impediment to the entrance to the secretion apparatus,^5^ or as a gatekeeper that determines substrate hierarchy.^6^ Across bacterial species, this protein (known as MxiC in *Shigella* sp.) possesses only weak sequence homology and in some species exists as two separate polypeptide chains (e.g., in *Yersinia* sp. the homologue consists of YopN and TyeA).^7^ Despite this, distinct functional homologies can be identified across species. Functional knock-outs of members of this family have no effect on needle formation or stability but significantly reduce or abolish the secretion of translocators.^8–11^ In addition, in several species these mutations also result in enhanced secretion of effector proteins.^9–14^ This differential effect on translocator and effector secretion suggests that these proteins have a role in T3SS discrimination between secreted proteins involved in translocation and proteins that have effector function.

There are, however, several differences between the members of this family. Most notably, activation of type 3 secretion in *Yersinia* sp. results in the secretion of YopN, while TyeA remains in the bacterial cytoplasm.^15,16^ The dissociation of YopN and TyeA has been proposed as a mechanism for the regulation of secretion but clearly cannot be a conserved mechanism in those species where the homologue is a single polypeptide chain.^17^ In the crystal structure of the *Y. pestis* YopN-TyeA complex, the close proximity of the C terminus of YopN with the N terminus of TyeA suggested that a single polypeptide encoding both proteins could maintain the same overall structure.^5^ In order to confirm this, we have determined and refined the structure of the *S. flexneri* homologue, MxiC, in three distinct crystal forms. The molecular architecture and movement of the domains of MxiC compared with that of YopN-TyeA highlights conformational differences that may play an important role in T3SS signaling.

## Proteolytic susceptibility of the N terminus of MxiC

**Fig. 1:**
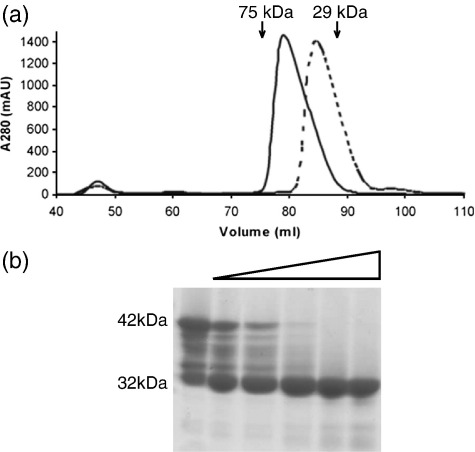
Size-exclusion chromatography and limited proteolysis of MxiC. a, Elution of MxiC_FL_ (continuous line) and MxiC_NΔ73_ (broken line) from a HiLoad 16/60 Superdex 200 column pre-equilibrated in 20 mM Tris (pH 7.5), 150 mM NaCl. MxiC_FL_ and MxiC_NΔ73_ elute as monomers as single, slightly asymmetric peaks. b, SDS-PAGE of limited proteolysis of MxiC_FL_. Degradation of purified MxiC_FL_ was considerable after storage at 4 °C for eight weeks (lane 1). Limited proteolysis was carried out on freshly purified MxiC_FL_ incubated for 2 h at 20 °C with an increasing mass ratio of protein:subtilisin from 20 μg:2 ng to 20 μg:80 ng (lanes 2–6). Methods: DNA fragments of the *mxiC* gene encoding residues 1–355 (full length, MxiC_FL_) and 74–355 (N-terminal truncation, MxiC_NΔ73_) were produced by PCR (FLf, CATATGCTTGATGTTAAAAATACAGGAGTTTTT; N73f, CATATGAGTCAGGAACGTATTTTAGAT; FLr, GAATTCTTATCTAGAAAGCTCTTTCTTGTATGCACT) and cloned into the NdeI-EcoRI sites of the pET28b vector. These constructs include an N-terminal His_6_-tag and a thrombin cleavage site. MxiC constructs were expressed in *Escherichia coli* BL21 (DE3) cells grown in LB medium containing 34 μg ml^− 1^ kanamycin. Cells were grown at 37 °C until an *A*_600_ nm of ∼ 0.6 was reached, whereupon they were cooled to 20 °C and protein over-expression was induced by the addition of IPTG (1.0 mM final concentration). After ∼ 16 h, cells were harvested by centrifugation (15 min, 5000***g***, 4 °C) and pellets were frozen at – 80 °C. Cell pellets were resuspended in lysis buffer (20 mM Tris (pH 7.5), 500 mM NaCl and Complete EDTA-free Protease Inhibitor Cocktail, Roche) and lysed using an Emulsiflex-C5 Homogeniser (Glen Creston, UK). The resultant cell suspension was centrifuged (20 min, 20,000***g***, 4 °C) and the soluble fraction was applied to a pre-charged HisTrap FF nickel affinity column (GE Life Sciences). Protein was eluted using a gradient of 0–1 M imidazole in 20 mM Tris (pH 7.5), 500 mM NaCl and fractions containing MxiC were further purified by size-exclusion chromatography as described above. SDS-PAGE analysis revealed MxiC_FL_ and MxiC_NΔ73_ to be pure (data not shown). Fractions containing purified MxiC were pooled and concentrated using Millipore Ultra-15 10 k MWCO centrifugal filtration devices to 7 mg ml^− 1^ and stored at 4 °C. Selenomethionine (SeMet)-labeled MxiC was produced by expression in the *E.coli met*^−^ auxotrophic strain B834 (DE3). Cultures were grown in LB medium to an *A*_600 nm_ of 0.9 then pelleted (15 min, 4000***g***, 4 °C) and washed in PBS three times before being used to inoculate SelenoMet Medium Base™ containing SelenoMet Nutrient Mix™ (Molecular Dimensions). Cells were grown and induced as described above. SeMet-labeled protein was purified as described above. Full incorporation of selenomethionine was confirmed by mass spectrometry. Dynamic light-scattering experiments were performed on a Viscotek model 802 DLS instrument using the OmniSIZE 2.0 acquisition and control software according to the manufacturer's instructions at 20 °C on a 1 mg ml^− 1^ protein sample in 20 mM Tris (pH 7.5), 150 mM NaCl.

A full-length construct of MxiC (residues 1–355, MxiC_FL_) was purified by nickel-affinity chromatography followed by size-exclusion chromatography and revealed that MxiC_FL_ elutes at a volume less than that of a monomer, but greater than that of a dimer (Fig. 1a). This result, combined with dynamic light-scattering data that revealed a major species with a larger than expected hydrodynamic radius (*R*_h_ ∼ 3.8 nm), suggested that MxiC_FL_ does not possess a globular structure and may possess an elongated fold and/or be partially disordered. In crystallization trials, MxiC_FL_ initially yielded three different crystal forms (two distinct *P*2_1_ and one *P*4_3_2_1_2) that diffracted only to 3.5–3.9 Å resolution. Selenomethionine-labeled MxiC_FL_ yielded crystals (*P*4_3_2_1_2 with a different cell) that diffracted to 3.2 Å resolution and were used for phasing by the multiple-wavelength anomalous diffraction (MAD) method (Table 1). Preliminary model building into these maps revealed that the first 70–80 residues were poorly ordered and not visible in the electron density. In addition, we observed that, in solution, the N terminus was susceptible to proteolytic degradation (Fig. 1b). Limited proteolysis with subtilisin followed by N-terminal sequencing and mass spectrometry revealed several degradation products resulting from cleavage of the N terminus up to residue 64. This proteolytically sensitive region of MxiC is equivalent to the region of YopN (32–76) that was shown to bind its chaperone and was disordered in the absence of this chaperone.^5^ Furthermore, *in vivo*, the stable expression and efficient secretion of YopN requires its chaperone.^18^ The proteolytic susceptibility of the N-terminal region of MxiC suggests that chaperone binding may act to protect MxiC from degradation via a similar mechanism. To date, a chaperone for MxiC has not been identified. In order to improve the quality of MxiC crystals, a shortened construct encompassing residues 74–355 (MxiC_NΔ73_) was expressed, purified (Fig. 1a) and subjected to crystallization trials.

## The structure of MxiC

**Fig. 2:**
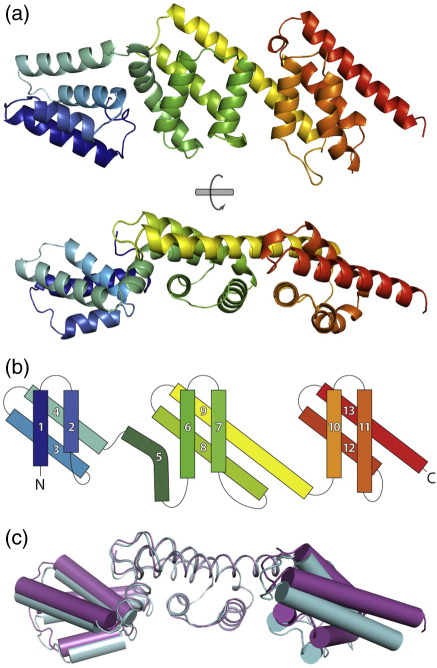
The structure and topology of MxiC. a, A ribbon diagram of MxiC, colored from blue at the N terminus to red at the C terminus. Views rotated by 90° about the long axis are shown. b, A diagram of the topology of MxiC illustrating the four-helix X-bundle of each domain colored as for a. c, Two molecules of MxiC from the *P*2_1_2_1_2_1_ crystal form (molecule B in magenta and molecule C in cyan), overlaid via their central domain (residues 154–265), illustrating the extremes of the movement seen for domains 1 and 3 (shown with cylindrical helices). Methods: Initial crystallization conditions were obtained by sparse-matrix screening,^30^ using the sitting drop vapor diffusion technique. Drops were prepared using an OryxNano crystallization robot (Douglas Instruments) by mixing 0.2 μl of protein (7 mg ml^− 1^ in 20 mM Tris (pH 7.5), 150 mM NaCl) with 0.2 μl of reservoir solution and were equilibrated against 100 μl of reservoir solution at 20 °C. Initial, low-resolution diffracting crystals of MxiC_FL_ grew within two weeks in condition P2-26 of the PACT Premier screen (0.2 M NaBr, 0.1 M BisTris–propane (pH 7.5), 20% (w/v) PEG3350: space group *P*4_3_2_1_2 with one molecule in the asymmetric unit) and condition 3 of Molecular Dimensions Structure Screen II (2% (v/v) dioxane, 0.1 M bicine (pH 9.0), 10% (w/v) PEG20000: two different, related *P*2_1_ forms with two molecules in the asymmetric unit). The former condition yielded diffraction-quality crystals of SeMet-labeled MxiC_FL_†. Crystals of native MxiC_FL_ diffracting to 3.0 Å resolution grew in 0.2 M Na_2_SO_4_, 0.1 M BisTris–propane (pH 6.5), 20% (w/v) PEG3350, again in *P*4_3_2_1_2 but with a longer *c* axis and two molecules in the asymmetric unit. The methylation reaction was performed as described in Refs. 31 and 32 on purified MxiC_FL_ and MxiC_NΔ73_ each at 1 mg ml^− 1^ in 50 mM Hepes (pH 7.5), 250 mM NaCl. Samples were centrifuged (5 min, 13,000 rpm, 10,000*g* 4 °C) before purification of soluble methylated protein by size-exclusion chromatography (as described above). Methylation of all lysine side chains and the N terminus was verified by mass spectrometry (42,952 Da for MxiC_FL_ and 35,106 Da for MxiC_NΔ73_). The *P*222 crystal form grew in 1.0 M succinic acid, 0.1 M Hepes (pH 7.0), 1% (w/v) PEG2000MME. The *P*2_1_2_1_2_1_ crystal form grew in 0.2 M sodium acetate, 0.1 M BisTris–propane (pH 7.5), 20% (w/v) PEG3350. Crystals of MxiC were cryoprotected in reservoir solution containing 25% (v/v) glycerol for 15 s and flash cryocooled in liquid nitrogen for data collection. Diffraction data were recorded at 100 K. Data were indexed and integrated in MOSFLM,^33^ and scaled with Scala,^34^ within the CCP4 program suite,^35^ except for the native MxiC_FL_*P*4_3_2_1_2 3.0 Å dataset, which was indexed in Labelit^36^ and integrated in XDS,^37^ both run from the processing suite Xia2 (G. Winter *et al.*, unpublished program). Initial phases were computed using SHARP:^38^ five sites were found by SHELXD^39^ run from the suite of programs autoSHARP^40^ against F_A_s calculated from the peak, inflexion and low-energy remote wavelengths of a SeMet-labeled *P*4_3_2_1_2 MxiC_FL_ crystal. The coordinates and *B*-factors of these sites were refined in SHARP against the above data plus the second remote wavelength from the same SeMet crystal. Solvent flattening was performed using CCP4-DM^41^ and SOLOMON,^42^ yielding a 3.5 Å map that was used for initial model building guided by the YopN–TyeA structure (PDB ID ***1xl3***).^5^ After alternate cycles of model building in Coot,^43^ refinement in Buster-TNT,^44^ and simulated annealing in PHENIX,^45^ this initial model was used for molecular replacement, using CCP4 PHASER,^46^ into the higher resolution *P*2_1_2_1_2_1_ form. The resultant model was used for molecular replacement against the MxiC_NΔ73_*P*222 and native MxiC_FL_*P*4_3_2_1_2 crystal forms. The final Buster-TNT refinements in the latter forms used NCS restraints throughout, and extra geometry restraints tying the geometry to Refmac^47^-refined models, to improve the stereochemistry (as Refmac5 implements torsion angle restraints and can refine riding H atoms), a refinement strategy devised by Dr. Stephen Graham (University of Oxford).

Reductive methylation of MxiC_NΔ73_ yielded crystals that diffracted to higher resolution: 2.85 Å and 2.5 Å in space groups *P*2_1_2_1_2_1_ and *P*222, respectively (Table 1). MxiC is an elongated rod-shaped molecule with a long axis of 86 Å (Fig. 2a). It is composed of three domains, each possessing a four-helix X-bundle fold (Fig. 2b).^19^ The first and last domains consist only of the X-bundle motif while the central domain also possesses a bent helix (α5) that is packed against domain 1. It is the first two domains of MxiC that are equivalent to YopN while the third domain of MxiC, equivalent to TyeA, is connected to domain 2 via a ten-residue linker. This linker acts only to tether the domains and does not have any major structural role, allowing the equivalent regions of MxiC and YopN–TyeA to adopt similar folds. There are a total of seven independent MxiC molecules in the crystallographic asymmetric units of the three refined crystal forms (see Table 1). These structures reveal that, although the fold of each domain is maintained in all structures (rmsd over C^α^ atoms of domains 1, 2 and 3 are 0.5 Å, 0.9 Å and 0.8 Å, respectively), there is some flexibility at the interfaces between domains resulting in a “wobble” of the terminal domains about the central domain (rmsd over all C^α^ atoms of 1.4 Å; Fig. 2c). The elongated shape of MxiC means that the most distal regions undergo the greatest displacement while the more central interdomain interfaces undergo minimal change.

The structural similarity between the domains of MxiC suggests that they might be the product of gene duplication events. Such internal repetition arising via intragenic duplication and recombination events has been a successful stratagem throughout evolution for enlargement of the available surface area.^20^ This, combined with the elongated shape of MxiC, provides the maximal exposure of surface area and considerable binding interfaces suitable for large substrates, linear peptides or multiple partners. This feature is typical of scaffolding proteins that act to recruit multiple proteins and enhance signaling.^21,22^

**Fig. 3:**
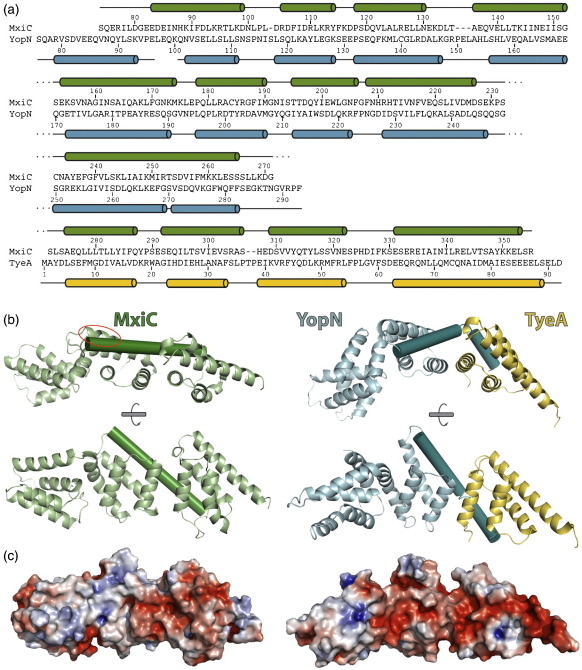
Comparison of MxiC with the YopN–TyeA complex. a, Structure-based sequence alignment of MxiC and YopN–TyeA. The positions of helices for each protein are illustrated (cylinders) above and below the sequence. b, Ribbon diagrams of MxiC (green) and YopN (cyan) complexed with TyeA (yellow). Helix α9 of MxiC and the equivalent helix of YopN are shown as cylinders. Two orientations rotated by 90° about the long axis are shown. The conserved hydrophobic patch on MxiC consisting of residues Leu222, Met226, Gly239, Leu242 and Leu245 is circled in red. c, Electrostatic surfaces of MxiC (left) and YopN-TyeA (right) are shown in the same orientation as in b (lower panel). Electrostatic surfaces were calculated using the APBS^48^ plugin of PyMol [http://www.pymol.org] with default settings. The electrostatic potential is displayed on the molecular surface and plotted in a red-white-blue scale (red, negative; blue, positive).

Pallen *et al.* used multiple sequence alignments to identify members of the MxiC/YopN–TyeA family from divergent Gram-negative species;^7^ a structure-based sequence alignment of MxiC and YopN–TyeA (Fig. 3a) differs in the N-terminal region from the alignment obtained in that work, and highlights the need to be cautious when interpreting conservation based on sequence alignments alone. The combination of structural and sequence alignment information identifies that most of the highly conserved residues are involved in maintaining the correct fold of these proteins. In support of this, mutations of YopN that constitutively block secretion replace highly conserved residues that are buried in the interfaces between domains of YopN.^23^ Ferracci *et al.* suggested that these mutations are likely to cause part of YopN to become unfolded or less well-ordered.^23^ Interestingly, mapping of sequence conservation onto the structure across the more closely related *Salmonella*, *Burkholderia* and *Shigella* species reveals a hydrophobic patch on the surface of the central domain consisting of residues Leu222, Met226, Gly239, Leu242 and Leu245 (Fig. 3b, red circle). This region is buried by crystal contacts in all seven independent molecules of MxiC in the *P*2_1_2_1_2_1_, *P*222 and P4_3_2_1_2 crystal forms, suggesting that this region may be a “hot spot” for protein–protein interactions.

A structural homology search using the DALI algorithm,^24^ and the Secondary Structure Matching algorithm of MSDfold,^25^ was unable to identify any protein, other than YopN, possessing multiple X-bundle motifs. However, two classes of proteins were identified that possess X-bundle motifs similar to the individual domains of MxiC. The structural homologues belonged to the Bcl-2 family of apoptosis regulators, specifically the *Caenorhabditis elegans* homologue Ced9 (DALI *Z*-score 4.3, **1ohu**)^26^ and the programmed cell death proteins involved in inhibition of protein synthesis, specifically the MA3 domain of Pdcd4 (DALI *Z*-score 3.9, **2nsz**).^27^ Although the significance of these similarities is uncertain, structural mimicry of apoptotic factors is a common theme seen in viral and bacterial pathogens and represents an important defense against the host immune response.^28,29^ This structural similarity may be of functional relevance, as MxiC has been identified as a protein that is secreted by the T3SS under conditions that mimic those encountered by bacteria during infection of a host.^3^

## Comparison with the YopN–TyeA complex

Although the individual domains of MxiC and YopN–TyeA adopt similar folds (rmsd over C^α^ atoms of the first and second domains of MxiC with the equivalent regions of YopN of 3.9 Å and 3.2 Å, respectively, and the third domain of MxiC with TyeA of 0.8 Å), the arrangement of these domains results in a different overall shape for these molecules (Fig. 3b). This structural rearrangement differs from the “wobble” seen between different MxiC molecules and is particularly noticeable in one orientation (Fig. 3b, top panel). This view highlights the straight conformation adopted by MxiC and the relative curvature of the YopN–TyeA complex. The different position and orientation of the first domain of MxiC may be due, in part, to the missing N-terminal portion, as the YopN–TyeA structure possesses an additional helix at its N terminus.

It seemed likely that the major difference between these structures would be the arrangement of the domain equivalent to TyeA as this is a separate polypeptide in the *Yersinia* structure. Surprisingly, the most striking difference instead involves the long á-helix (α9) in the central domain of MxiC, which is straight in all of our MxiC structures, but possesses a sharp kink in YopN when complexed with TyeA (Fig. 3b). It is the straightening of this helix that results in a reorientation of the C-terminal domain of MxiC, compared with TyeA, and opens one face of the molecule. As the sequence at the hinge is not conserved, and all MxiC molecules possess the straight helix conformation, these structures may represent genuine differences between species. Alternatively, it may represent a conformational switch that, in this case, was captured due to the very different pH for the YopN–TyeA structure (pH 10.5) compared with the MxiC structures (pH 6.5–7.5).

Despite the lack of sequence conservation on the surface of these structures, an analysis of the surface electrostatics reveals that the face that is curved in YopN–TyeA and open in MxiC possesses a conserved negatively charged patch (Fig. 3c). This patch spreads across one face of the C-terminal half of the molecule (displayed in red on the right-hand side of the surface shown in Fig. 3c). The helices that line this face undergo minor repacking and can accommodate the large movements of the surrounding domains. The conserved patch on this face suggests a role for this region in interactions with partner proteins. If this is a binding face for another component of the T3SS, it is interesting to note that this interface is conserved despite it being intramolecular in one homologue and intermolecular in another.

## Protein Data Bank accession numbers

The atomic coordinates and structure factors have been deposited at the RCSB Protein Data Bank with accession codes 2vix (*P*2_1_2_1_2_1_), 2vj4 (*P*222) and 2vj5 (*P*4_3_2_1_2).

## Figures and Tables

**Table 1:** Statistics for crystallographic data collection and structure refinement (values in parentheses are for the highest resolution shell)

	MxiC_NΔ73_ methylated	MxiC_NΔ73_ methylated	MxiC_FL_	MxiC_FL_ SeMet			
A. *Data collection*				Peak	Inflexion	Rm1	Rm2
No. crystals used	1	1	1	1			
X-ray source	ESRF ID29	Diamond I03	ESRF ID29	ESRF ID29			
Detector	ADSC CCD scanner	ADSC CCD scanner	ADSC CCD scanner	ADSC CCD scanner			
Wavelength (Å)	0.9756	0.9757	0.9760	0.9799	0.9801	1.033	0.9756
Space group (*Z*)	*P*222 (8)	*P*2_1_2_1_2_1_ (12)	*P*4_3_2_1_2 (16)	*P*4_3_2_1_2 (8)			
Unit-cell dimensions							
*a* (Å)	83.48	89.31	91.37	85.54			
*b* (Å)	83.45	102.97	91.37	85.54			
*c* (Å)	117.07	123.57	215.84	118.2			
Resolution limits (Å)	42.0–2.5 (2.64–2.50)	50.4–2.85 (3.00–2.85)	38.6–3.0 (3.16–3.00)	33.0–3.5 (3.69–3.50)	30.3–3.6 (3.79–3.60)	30.3–3.6 (3.79–3.60)	32.22–3.7 (3.90–3.70)
Measured reflections	100,493 (14,918)	84,697 (9,365)	129,136 (18,976)	49,227 (7385)	69,519 (10,267)	69,324 (10,270)	42,578 (6366)
Unique reflections	28,754 (4173)	26,409 (3546)	18,797 (2675)	5964 (848)	5539 (773)	5525 (773)	4982 (714)
Completeness (%)	99.4 (99.9)	98.6 (93.3)	98.5 (98.4)	99.9 (99.9)	99.6 (99.9)	99.8 (99.9)	98.3 (99.3)
Multiplicity	3.5 (3.6)	3.2 (2.6)	6.9 (7.1)	8.3 (8.7)	12.6 (13.3)	12.5 (13.3)	8.5 (8.9)
*R*_merge_	0.068 (0.518)	0.112 (0.490)	0.072 (0.434)	0.136 (0.453)	0.135 (0.526)	0.142 (0.533)	0.140 (0.487)
*R*_pim_	0.035 (0.269)	0.060 (0.283)	0.027 (0.164)	0.047 (0.153)	0.039 (0.144)	0.040 (0.145)	0.047 (0.160)
Average *I*/σ(*I*)	14.2 (2.7)	10.7 (2.4)	14.0 (4.5)	17.5 (4.9)	20.3 (5.3)	20.4 (5.4)	16.3(4.5)
Wilson *B*-value (Å^2^)	59.3	65.5	171	79.5	59.0	55.3	62.7
B. *Refinement*				C. *MAD Phasing statistics*			
Resolution Range (Å)	42.0–2.5 (2.65–2.50)	50.4–2.85 (3.00–2.85)	38.6–3.0 (3.18–3.0)	SHARP FOM_acentrics_: 0.945 (32–14 Å); 0.491 (32–3.5 Å); 0.174 (3.6–3.5 Å)			
Working set reflections	23,449 (3,933)	25,054 (3,693)	17,728 (2,762)	SHARP FOM_centrics_: 0.789 (32–14 Å); 0.395 (32–3.5 Å); 0.127 (3.6–3.5 Å)			
Free set reflections	1259 (217)	1314 (211)	1014 (154)				
*R*	0.211 (0.237)	0.244 (0.245)	0.246 (0.306)	SHARP phasing power (iso/ano):			
*R*_free_	0.265 (0.278)	0.273 (0.295)	0.270 (0.375)	Peak: (-)/1.3; Inflexion: 1.3/0.1; Rm1:0.4/0.9;Rm2:0.3/0.6			
Residues	A/B:64-355	A:73-355	A/B:73-355	Solvent flattened FOM_overall_: 0.897 (32–8 Å); 0.870 (32–3.5 Å); 0.799 (3.6–3.5 Å)			
		B:73-355					
		C:72-352					
Protein atoms	4776	6967	4750				
Water molecules	191	135	13				
r.m.s.d. from ideal							
Bond lengths (Å)	0.006	0.006	0.005				
Bond angles (deg.)	0.935	0.821	0.773				
Mean protein *B*-factor (Å^2^)	62.8	60.0	140				
Ramachandran plot (non-Gly and Pro), residues in							
Favored regions (%)	94.4	95.8	92.2				
Allowed regions (%)	97.6	99.5	98.4				
PDB ID	2VJ4	2VIX	2VJ5				

## Footnotes

† Neither the structures of the crystal forms diffracting at low resolution nor the SeMet *P*4_3_2_1_2 form were fully refined nor are reported in Table 1.
